# Book Notices

**Published:** 1897-06

**Authors:** 


### Book Notices.

The April (1897) Number of The Alienist and Neurologist contains: "Psychical Hermaphroditism," "A Few Notes on Sexual Perversion, with Two Clinical Cases of Sexual Inversion," by Wm. Lee Howard, M. D., Baltimore Md.; "Preputial Reflex Epileptiform Convulsions, with Report of a Case," by Alex. L. Hodgson, M. D.; "On Intemperance, Consanguine Marriages and Educational Overpressure, as Factors in the Genesis of Nerve Disease and Degeneration of the Race," by Sir Frederic

Bateman, M. D., LL. D., F. R. C. P.; "What is Meningitis?" by W. S. Christopher, M. D., Chicago, Ill.; "The Case of Sturgeon Young," "A Question of Hypnotic Injury and Death," by Clark Bell, Esq.; "Encephalitic and Late Epilepsy," by Jas. G. Kiernan, M. D., Chicago; "Psychoses of Old Age," by Harriet C. B. Alexander, A. B., M. D., Chicago, Ill.; "The Auto-Toxic Origin of Epilepsy," by J. Nelson Teeter, M. D.; besides the usual selections, editorials, reviews, book notices, etc. Subscription, $5 per annum; single copies, $1.50.

C. H. Hughes, M. D., Editor, 3827 Olive Street, St. Louis, Mo.

---

A Manual of Venereal Diseases. By James R. Hayden, M. D., Chief of Venereal Clinics at the College of Physicians and Surgeons (Columbia University), New York; Professor of Genito-Urinary and Venereal Diseases in the Medical Department of the University of Vermont; Visiting Physician to the New York City Hospital. In one volume of 267 pages, with 47 illustrations. Price in cloth $1.50. Lea Brothers & Co., Publishers, New York and Philadelphia, 1896.

The book is divided into three parts, Part I treating of gonorrhea and its complications; Part II of chancroid, and Part III of syphilis. The discussion of these different diseases, their etiology, pathology, diagnosis and treatment, while concise, will be found very satisfactory to the medical student and the busy physician. The author makes the best use of the space, devoting it mainly to the clear presentation of practical points in the management of cases suffering from these diseases. S. E. H.

---

Twentieth Century Practice. An International Encyclopedia of Modern Medical Science. By Leading Authorities of Europe and America. Edited by Thomas L. Stedman, M. D., New York City. In Twenty Volumes. Volume IX. "Diseases of the Digestive Organs." New York: William Wood & Company. 1897.

The contributors to this volume are C. A. Ewald, M. D., Berlin; Kendal Franks, M. D., Johannesburg, S. A. Republic; V. P. Gibney, M. D., New York; Carlo Gioffredi, Naples; Werner Kuemmel, M. D., Breslau; Johann Mikulicz, M. D., Breslau; John B. Murphy, M. D., Chicago; Mariano Semmola, M. D., Naples; Alfred Stengel, M. D., Philadelphia, and John B. Walker, M. D., New York.

The opening chapter is on local diseases of the mouth, by Drs. Mikulicz and Kuemmel. Dr. Ewald's contribution on diseases of the intestines (excluding infectious diseases, parasites, and hernia,) covers about 180 pages. Hernia is the subject of an article by Drs. Gibney and Walker. Dr. Alfred Stengel writes on diseases of the spleen. The longest article in the volume is the one by Drs. Semmola and Gioffredi on diseases of the liver. This article covers 325 pages. Dr. Murphy writes on diseases of the gall bladder, and Dr. Franks on movable kidney. The subjects discussed in this volume are important ones, and the physician who has the book in his library will find much satisfaction in referring to it when he wishes a full and up-to-date discussion by the very best authors. S. E. H.

